# Supplementary material for: Automaticity in processing spatial-numerical associations: Evidence from a perceptual orientation judgment task of Arabic digits in frames
Source: PLoS One. 2020 Feb 24;15(2):e0229130. doi: 10.1371/journal.pone.0229130 (PMC7039522; doi:10.1371/journal.pone.0229130)
Supplement: Attachment — Submitted filename: Response to Reviewers2.docx [file pone.0229130.s007.docx]

Review Comments to the Author

Please use the space provided to explain your answers to the questions above. You may also include additional comments for the author, including concerns about dual publication, research ethics, or publication ethics. (Please upload your review as an attachment if it exceeds 20,000 characters)

Reviewer #1: I'd like to thank the authors for their thorough and well-written cover letter. They have done a good job of responding to the reviewers' comments and I wish them luck with this line of research.

I am happy to see the work published as it is now - I just have one very minor point of clarification. I think line 541 (the version without track changes) has a minor error. I think that "non-symbolic numerosities (1-9 circles) in an orientation decision task" should read "non-symbolic numerosities (1-9 triangles) in an orientation decision task". The paper cited does use circles for color decision, but I believe the orientation task used triangles - perhaps the authors could double-check this.

*Response: Thank you so much for your comments and correction. We corrected this mistake.*

p. 22, l. 540 - 543:

Consistent with these accounts, previous studies using non-symbolic numerosities (1-9 triangles) in an orientation decision task also revealed a stronger SNARC in 1-4 compared to 6-9, indicated a more precise spatial association in a smaller number range [35].

Reviewer #2: I commend the authors as they have answered all my concerns, I think that the manuscript makes a nice contribution.

*Response: Thank you so much for your comments.*
